# Malignant adnexal tumor of the skin on breast: A case report of apocrine carcinoma

**DOI:** 10.1016/j.ijscr.2023.108383

**Published:** 2023-06-02

**Authors:** Putu Anda Tusta Adiputra, I Wayan Sudarsa, Hendry Irawan, Herman Saputra

**Affiliations:** a Surgery Department, Surgical Oncology Division, Faculty of Medicine, Udayana University, Denpasar, Bali, Indonesia; b Anatomical Pathology Department, Faculty of Medicine, Udayana University, Denpasar, Bali, Indonesia

**Keywords:** Malignant adnexal tumor, Breast, Apocrine carcinoma, Breast carcinoma, Treatment, Case report

## Abstract

**Introduction:**

Malignant adnexal tumors of the skin (MATS) are a group of rare and varied tumors that lack standardized guidelines for their management. Apocrine carcinoma (AC) is a highly uncommon form of breast malignancy, contributing to less than 1 % of all female invasive breast carcinomas. AC has a similar microscopic growth pattern to invasive ductal carcinoma, which can result in early misdiagnosis.

**Presentation of case:**

This report presents a case of a 67-year-old female with a lump in the superior lateral quadrant of her left breast for six years. Surgical therapy was performed with wide excision due to clinical operability, no significant involvement of the axillary lymph nodes, and without metatasis. During the operation, Wide excision of 1–2 cm free margin according to standard and local reconstruction flaps were performed, with berry packing for the identified lymph nodes.

**Discussion:**

The tumor was ER and PR negative, so hormonal treatment would be ineffective, assuming that this is an apocrine carcinoma of the breast. A metastatic work up was already done, and no metastasis was found. A mastectomy would appear to be a viable option.

**Conclusion:**

It is important to perform a clinical reevaluation to provide optimal treatment for breast malignancy. Misdiagnosis can occur early. In this case, a surgical procedure involving wide excision was performed, and as of now, the patient has not reported any recurrence.

## Highlights

• Malignant adnexal tumors of the skin (MATS) are uncommon yet aggressive and sometimes misdiagnosed as breast carcinoma.
• MATS, in this case, has characteristics of the immunohistochemistry as TNBC, and the pathology report shows adenocarcinoma showing apocrine-like cell differentiation.
• Effective operative management, can result in the absence of cancer recurrence.

## 1 Introduction

Malignant adnexal tumors of the skin (MATS) are a rare and diverse group of tumors with no standardized guidelines for their management. MATS can originate from various gland types (eccrine, apocrine, sebaceous, sweat duct or ceruminous) within the skin or follicular cells, resulting in different histologic entities [1]. Carcinoma of the apocrine gland (AC) is an uncommon form of breast malignancy, accounting for less than 1 % of female invasive breast carcinomas. Despite limited information in the literature, AC presents a risk of recurrence. Apocrine carcinoma may be identified by the appearance of apocrine cells within the tumor, and it is considered a type of invasive ductal breast cancer. Similar to other invasive ductal cancers, apocrine breast cancer typically arises from the milk duct of the breast and then spreads to surrounding tissues. The cells of an apocrine tumor differ from those of a typical ductal cancer [2,3].

From the aspect of microscopical appearance, carcinoma of the apocrine gland shares the same growth pattern as invasive ductal carcinoma, not otherwise specified (IDC-NOS), but differs in the appearance of its cells. The cells of apocrine carcinoma exhibit prominent eosinophilic granular cytoplasm and multiple nucleoli, which are typical of apocrine cells. These cells resemble those found in the sweat glands of the underarm and groin regions. It is believed that normal breast ductal cells undergo metaplasia, or a change in form, to become more like apocrine cells. However, the exact cause of this transformation is not yet fully understood [4]. Recent research indicates that apocrine carcinomas generally lack estrogen and progesterone receptors, but have androgen receptor positivity (ER-, PR- AR+) and express the protein Gross cystic disease fluid protein-15 (GCDPF-15).

Misdiagnosis can occur early in this case, so the patient underwent chemotherapy to several countries, including Malaysia and alternative therapy in South America. There was no detailed information regarding the chemotherapy that was received by the patients. The patient receives several clinical evaluation, diagnostic, and surgery in Bali, Indonesia. This disease requires regular follow-up of treatment, but during the pandemic there are limitations in treatment so that it becomes an obstacle for patients to continue treatment. In the context of apocrine breast cancer, local therapy aims to prevent breast cancer recurrence by performing either lumpectomy or mastectomy surgery.

This report was written in line with the SCARE criteria [5].

## 2 Presentation of case

A 67-year-old female presented with history of lump in the superior lateral quadrant of her left breast in the last six years. Retraction of the nipples and discharge were negative, with no abnormality detected on general physical examination. The left breast was apparently normal without any axillary lymphadenopathy. Previously, the patient had a surgical pathology report after excision of her lump biopsy in 2015 with a diagnosis of moderately differentiated infiltrating adenocarcinoma and immunohistochemical examination, the main source of malignancy could not be identified, positive malignant cells for CK7, CK19, E-Cadherin and MOC31 immunomarkers and TTF-receptor-1, CK20, CDX2, GCDFP-15, Mammoglobin Her2/Neu, Estrogen and Progesterone negative.

**Fig. 1:**
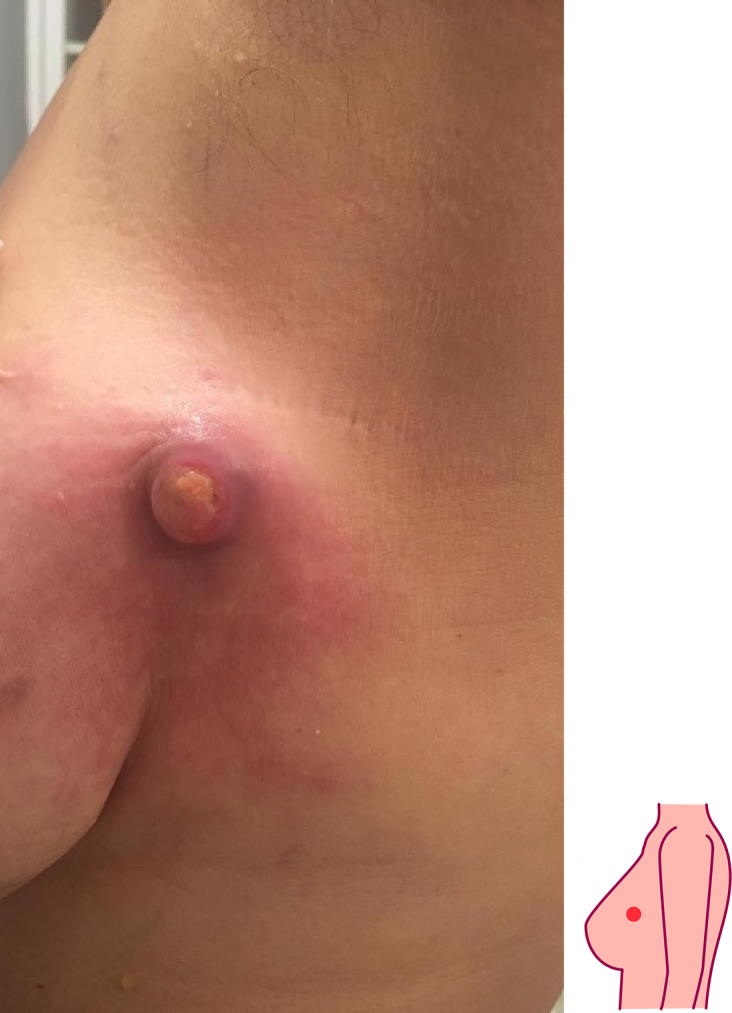
First clinical appearance.

The patient was firstly went to physician in 2015 in Boston, United States, but after the diagnostic procedure, patient refused to continue any medical procedure and chose to do the alternative medication in Peru. Patient stated that she had given chemotherapy twice in Malaysia on 2021 (Fig. 1), but she felt that the lump grew bigger, and she was devastated and traveled to Bali but she cannot went back to her country because of the COVID-19. Finally, she got herself a medical checkup in Bali, Indonesia.

**Fig. 2:**
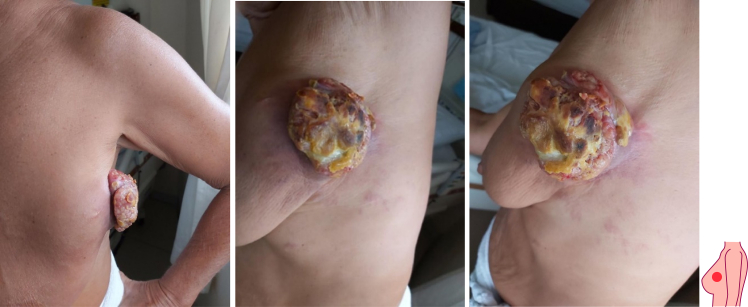
Clinical appearance after 3 months.

**Fig. 3:**
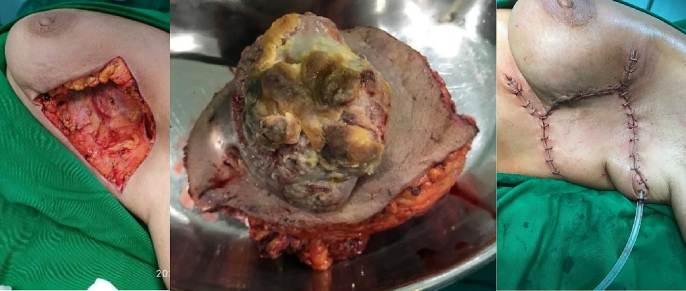
Wide excision the tumor and reconstruction.

**Fig. 4:**
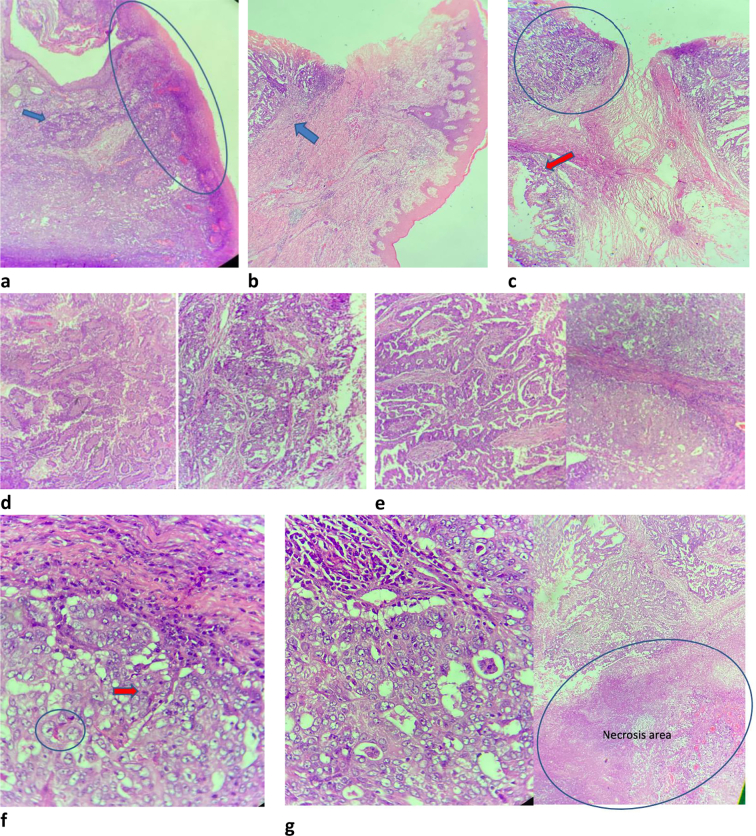
Microscopic anatomical pathology. (a) The specimen show epidermal layers with broad necroulcerative appearance (marked). Epidermal rete ridges foci involved by neoplastic cells can be observed, impression of epidermotropism of neoplastic cells (arrow). (b) The specimen from left breast consist of epidermal layers, and dermis, tumor mass show infiltrative & pushing border, involving the dermis (arrow). (c) Tumor mass show infiltrative & pushing border, involving the dermis. It consists of neoplastic epithelial cells with a complex arrangement, in the form of interconnecting patterns, tubular, cribriform (circled) & dilated structure (arrow) with papillary formation. (d) It consists of neoplastic epithelial cells with a complex arrangement, in the form of interconnecting patterns, tubular, cribriform & dilated structure with papillary formation. (e) Solid pattern with pushing broder (right), interconnecting patterns, tubular, cribriform (left side). (f) The individual neoplastic cells reveal well-defined border, enlarged - round - centrally located with prominent nucleoli & moderate - abundant slightly granular eosinophilic to pale vacuolated cytoplasm (marked). Mitosis can be observed (arrow).

In 2021, after re-evaluation, the lump considered clinically still operable without metastatic (Fig. 2), then we do the surgical therapy with wide excision of 1-2 cm free margin with local reconstruction flap to cover post operation defect and performed berry packing for the identified lymph nodes (Fig. 3). Pathological examination was carried out after surgery and the report found as Adenocarcinoma showed apocrine-like cell differentiation with a differential diagnosis of primary breast carcinoma (primary cutaneous adnexal carcinoma with apocrine differentiation, grade 2 according to Bloom Richardson Score, NST invasive ductal carcinoma with apocrine differentiation grade 2), metastatic adenocarcinoma from elsewhere to the breast (secondary carcinoma) (Fig. 4).

After receiving the surgical therapy, the patient returned to her country and got no other therapy. Patient contact is maintained, and currently, the patient is in the United States with no recurrence to date (about one year following the surgical intervention).

## 3 Discussion

In this case, the patient whom was a 67 year old female, was admitted to surgical intervention with wide excision and reconstruction. The lump was found to be on the superior lateral quadrant which had persisted during the past six years. Metastatic work up already done and found no metastatic in this patient. The pathology report was given as Adenocarcinoma showing apocrine like cell differentiation with differential diagnose as a primary breast carcinoma (primary cutaneous adnexal carcinoma with apocrine differentiation, grade 2 according to Bloom Richardson Score, invasive ductal carcinoma of NST with apocrine differentiation grade 2), metastatic adenocarcinoma from other site to breast (secondary carcinoma). Based on the Elston-Ellis modification of the Bloom and Richardson grading system then invasive apocrine carcinomas are usually categorized as grade 2 or 3 carcinomas. This grading system reflects the architectural pattern of the tumor cells, which indicates their level of differentiation towards normal breast ducts and lobules [1].

Vranic et al. have reported that Apocrine Carcinoma (AC) is a rare form of breast malignancies, constituting to only 0.3–4 % of all breast malignancies which often depend on criteria's that have been used to define it [2]. When defined strictly as above, it comprises approximately 1 % of female invasive breast carcinoma [3]. AC is an aggressive type of cutaneous adenocarcinoma that is more commonly seen in middle-aged women, and it tends to occur in regions of the body that are rich in apocrine glands, such as the axilla, as well as in other sites like the eyelid (in Moll's glands), ear, and scalp [4].

Macroscopic and clinical characteristics shows that apocrine carcinoma of the breast typically presents as a solidified whitish mass with a fine infiltrative line. Nipple discharge may or may not be present [3]. on microscopic examination, the apocrine carcinoma exhibits similar pattern for its growth as invasive ductal carcinoma, NST. This includes a rare encapsulated apocrine papillary carcinoma of the breast, which is clearly identifiable as a cyst which are well demarcated on microscopical examination. The cells of apocrine carcinoma are characterized by typical apocrine features, such as abundant eosinophilic granular cytoplasm and prominent/multiple nucleoli. These cells resemble those found in the sweat glands of the underarm and groin region. The transformation of normal ductal breast cells into apocrine cells is believed to occur through metaplasia, although the underlying mechanisms are not fully understood [2].

Invasive apocrine breast cancer is an infrequent form of breast cancer that, like other types of invasive ductal cancer, originating from the ducts of the breast which then spreads to the surrounding tissues. One of the risk factors for breast cancer is age, where this patient is 67 years old, which means that he has experienced menopause. Menopause after 55 years of age will be at risk of developing breast cancer because of an imbalance of the hormones estrogen and progesterone [6]. It is important to carry out clinical reevaluation with unusual or unusual pathological results so that mismanagement does not occur. The objective of local therapy for apocrine breast cancer is to minimize the likelihood of breast cancer recurrence by targeting the affected area of the breast. Local therapy typically includes surgical procedures such as lumpectomy or mastectomy [7].

Like in this patient, we found that the pathology result is adenocarcinoma showed apocrine-like cell differentiation. The malignant adnexal tumor is rarely found and especially the site in the breast skin can be misdiagnosed as other breast cancer or any other breast pathology. It is crucial to conduct clinical reevaluations with exceptional or unusual pathological results to avoid mismanagement.

The malignant cells are positive for CK7, CK19, E-Cadherin and MOC31 immunomarkers. CK7 and CK19 indicate invasive apocrine carcinoma [2,8], E-Cadherin positive in ductal carsinom [8] similar to this case that happened in breast, MOC31 for metastatic adenocarcinom [9] in accordance with the pathology report. Other markes, TTF-1, CK20, CDX2, GCDFP-15, Mammoglobin Her2/Neu, Estrogen and Progesteron receptors are negative.

According to some studies, apocrine carcinomas predispose to estrogen and progesterone receptor negativity, androgen receptor positivity (ER -, PR- AR +) and expression of Gross cystic disease protein fluid-15 (GCDPF-15) [2,3,10]. The expression of androgen receptors were found to be noticed in up to 80 % of breast malignancies including ER-/PR- breast carcinomas. Androgen receptors are known to be a member of the steroid receptor family. It is a single polypeptide with several capability to mediate different functions with one of them being implicated in breast cancer development and progression. AR exerts anti-estrogenic, growth-suppressive effects in normal breast tissue and ER-positive breast carcinomas. ER-/PR- shows promoting cell growth and tumor development [2,11]. GCDFP-15 is present in the liquid of breast cysts and any apocrine cells: mammary glands, salivary glands, sweat, Paget's disease, etc.

TTF-1 negative shows that absent of thyroid transcription factor 1 [12], CK20 negative means there is no metastatic to lung, breast, thyroid, pancreas, and female genital tract [13], also CDX2 is a biomarker of mature colon cells, was shown to predict metastatic carcinoma in colon [14]. This patient has no recurrence to date (about one year following the surgical intervention). This shown that malignant adnexal life survival of this patient still good until recently, even though the patient refused to received any radiotherapy and decided to go back to her county.

## 4 Conclusion

Malignant adnexal tumors of the skin (MATS) such as Apocrine carcinoma (AC) is an exceptionally uncommon form of breast malignancy which in this case a 67-year-old female, with a lump in the superior lateral quadrant of her left breast which had occurred since six years ago. Misdiagnosis can occur early in this case, as shown in the early surgical pathology immunohistochemistry showing TNBC. It is important to perform the clinical reevaluation to give the best treatment to prevent the malignancy from coming back to the breast. In this case, we perform the surgical therapy with wide excision and there is no recurrence to date reported by the patient.

## Funding source

There is no funding source.

## Declaration of competing interest

There is no conflict of interest.
